# Acute Kidney Injury in a Patient With Cryoglobulinemia Secondary to Hepatic Mucosa-Associated Lymphoid Tissue Lymphoma: Case Report and Literature Review

**DOI:** 10.7759/cureus.10451

**Published:** 2020-09-14

**Authors:** Artsiom Klimko, Georgiana A Toma, Nona Bejinariu, Silviu-Mihai Secareanu, Iuliana Andreiana

**Affiliations:** 1 Division of Physiology and Neuroscience, University of Medicine and Pharmacy "Carol Davila", Bucharest, ROU; 2 General Medicine, University of Medicine, Pharmacy, Science, and Technology of Târgu Mureş, Târgu Mureş, ROU; 3 Department of Pathology, Santomar Oncodiagnostic, Cluj-Napoca, ROU; 4 Department of Nephrology and Dialysis, "Dr. Carol Davila" Teaching Hospital of Nephrology, Bucharest, ROU; 5 Department of Internal Medicine and Nephrology, University of Medicine and Pharmacy "Carol Davila", Bucharest, ROU

**Keywords:** maltoma, mucosa-associated lymphoid tissue, lymphoma, cryoglobulinemia, membranoproliferative glomerulonephritis, renal involvement

## Abstract

We report a patient with IgM-predominant type I cryoglobulinemia (CG), who presented to our nephrology department with acute kidney injury. He was previously diagnosed with sensorimotor neuropathy, which was in remission with maintenance dose of corticosteroids. Upon admission, there were ulcerated, necrotic cutaneous lesions localized to the inner aspect of the thighs bilaterally. Further workup revealed a mucosa-associated lymphoid tissue lymphoma, causing type I CG. Screening tests for hepatitis viruses were negative at this time. Under treatment with diuretics and high-potency glucocorticoids, the patient had an acceptable recovery of renal function and was referred to oncology for treatment. Unfortunately, three months later the patient succumbed to fulminant hepatitis, presumably secondary to reactivation of an occult hepatitis B/D co-infection. We further conducted a literature review to better describe patient characteristics and renal involvement in type I CG.

## Introduction

Cryoglobulinemia (CG) is characterized by immunoglobulin (Ig), which reversibly precipitates at low temperature (<37°C). The most commonly used classification of CG was proposed by Brouet et al. in 1974 and divides this pathology into three types based on immunochemical properties of the precipitated Ig [1]. Type I cryoglobulins are formed by isolated monoclonal Ig, and etiologies are closely linked to a specific Ig fraction - immunoglobulin M (IgM) is associated with Waldenstrom macroglobulinemia (WM), while immunoglobulin G (IgG) can be caused by multiple myeloma (MM), monoclonal gammopathy of undetermined significance (MGUS), B-cell non-Hodgkin’s lymphoma (B-NHL), or leukemia. Type II and III cryoglobulins are also called mixed as the precipitating Ig is a mixture of mono- and polyclonal Igs, and over 80% of cases are driven by an infectious etiology - classically, chronic hepatitis C virus (HCV) [2].

The exact pathogenesis of CG is unclear and multifaceted, likely involving environmental triggers, polygenic host susceptibility, and aberrant autoantibody production [3]. Clinically, high levels of circulating Ig cause hyperviscosity, while aggregates of cryoprecipitate obstruct vasculature causing multisystemic ischemic lesions, as was seen in our patient [4]. Renal involvement usually manifests in the form of membranoproliferative glomerulonephritis (MPGN) and has a variable course. Our objective is to discuss renal involvement in type I CG through presenting a case of type I CG that was caused by a primary hepatic mucosa-associated lymphoid tissue (MALT) lymphoma and manifested with acute kidney injury (AKI), sensorimotor neuropathy, and ulcero-necrotic cutaneous lesions. 

## Case presentation

**Figure 1:**
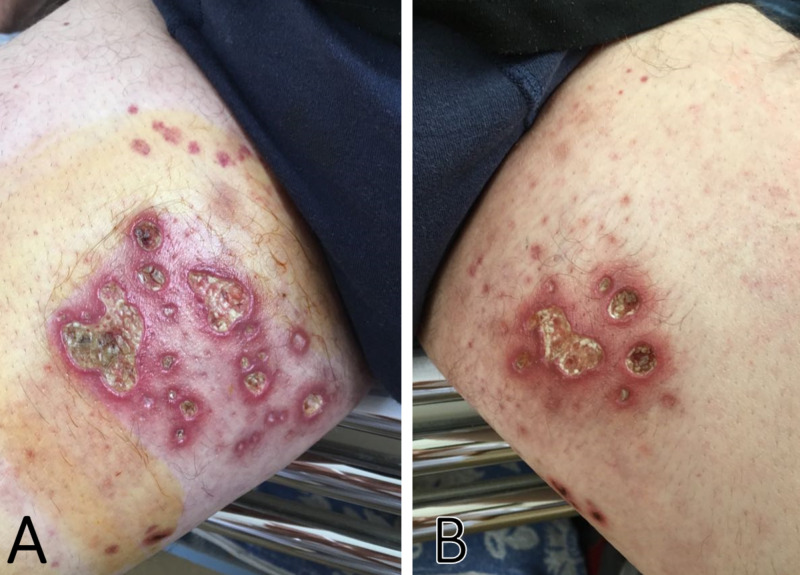
Cutaneous lesions with ulceration and necrosis that were present on the left (A) and right (B) legs of the patient upon admission.

A 58-year-old man presented to our nephrology department with chief complaints of oliguria, lower extremity edema, and resting dyspnea that had increase in severity over the past five days. A year earlier the patient was hospitalized for severe pain and paresthesia in the lower limbs, which led to the diagnosis of axonal sensorimotor polyneuropathy via nerve conduction studies (24.4% reduction in conduction velocity). Physical examination during this admission revealed an afebrile, jaundiced patient with several ulcero-necrotic lesions localized to the inner aspect of the thighs bilaterally (Figure 1). There was a dullness on percussion of the lower right hemithorax, with an absent vesicular sound at this level. Blood pressure was 140/90 mmHg, heart rate 70 beats per minute, no abnormal heart sounds were present, and there was a regular apex beat.

The patient’s lab results are presented in Table 1. Elevated creatinine (2.7 mg/dL versus 0.9 mg/dL that was noted one month prior) and urea confirmed AKI. Transaminases with conjugated bilirubin were moderately elevated in a background of negative serology for hepatitis B virus (HBV), HCV, and human immunodeficiency virus (HIV). IgM was almost double the upper limit of normal, while complement (C3/C4) was decreased. Rheumatoid factor, cryoglobulins, and serum viscosity were increased. Urinary light chain kappa concentrations were severely elevated. Serum protein electrophoresis revealed mild increases in alpha-1 globulins (0.3) with all other bands being within reference range. Critical for the workup of the cutaneous vasculitis, antineutrophil cytoplasmic antibodies and antinuclear antibodies were negative.

**Table 1:** Lab results at admission. WBC: white blood cells; RBC: red blood cells; HPF: high powered field; Ig: immunoglobulin; HBs: hepatitis B surface antigen; HCV: hepatitiv C virus; HIV: human immunodeficiency virus; DNA: deoxyribonucleic acid.

Lab values	Reference ranges	Patient values on admission
Complete blood count		
Hemoglobin (g/dL)	13-17.5	11.5
WBCs (x 10^9/L)	4-10	6.3
Platelet count (x 10^9/L)	150-400	96
Biochemistry		
Serum albumin (g/dL)	3.4-5.4	2.8
Serum protein (g/dL)	6-8	5.2
Urea (mg/dL)	8-21	131
Serum creatinine (mg/dL)	0.84-1.21	2.7
Uric acid (mg/dL)	4.0-8.5	15.0
Aspartate aminotransferase (U/L)	5-30	424
Alanine aminotransferase (U/L)	5-30	296
Gamma glutamyl transferase (UL)	6-50	105
Conjugated bilirubin (mg/dL)	<0.3	2.71
Total bilirubin (mg/dL)	0.1 to 1.2	4.59
Prothrombin time (seconds)	11-14	78
Immunology and inflammation		
IgA (g/L)	0.80-4.0	1.15
IgM (g/L)	0.50-2.0	5.2
IgG (g/L)	6.0-16.0	8.0
C3 (g/L)	0.08-0.16	0.67
C4 (g/L)	0.012-0.042	0.01
Fibrinogen (g/L)	1.8-4	43.4
C-reactive protein (mg/L)	<5	45
HBs antigen	Negative	Negative
Anti-HCV antibodies	Negative	Negative
HIV antibodies	Negative	Negative
Antinuclear antibodies	Negative	Negative (low titer)
Cytoplasmic anti-neutrophil antibodies (IU/mL)	≤1.9	0.03
Perinuclear anti-neutrophil cytoplasmic antibodies (IU/mL)	<3.5	0.11
Lactate dehydrogenase (U/L)	50-150	299
Anti-double stranded DNA (IU/mL)	<30.0	14
Rheumatoid factor (IU/mL)	<25	12
Cryoglobulins	NAs	+++
Serum viscosity (cP)	<1.5	1.8
Electrolytes		
Sodium (mg/dL)	135-145	130
Potassium (mg/dL)	3.5-5	4.15
Calcium (mg/dL)	8.5-10.2	4.0
Magnesium (mg/dL)	1.5-2	2.1
Phosphate (mg/dL)	3.4 to 4.5	5
Urinalysis		
Proteinuria (g/24h)	≤0.15	1.05
Kappa light chains	0.33-1.94	29.6
Lambda light chains	0.57-2.63	2.2
Kappa/Lambda report	0.26-1.65	13.3

**Figure 2:**
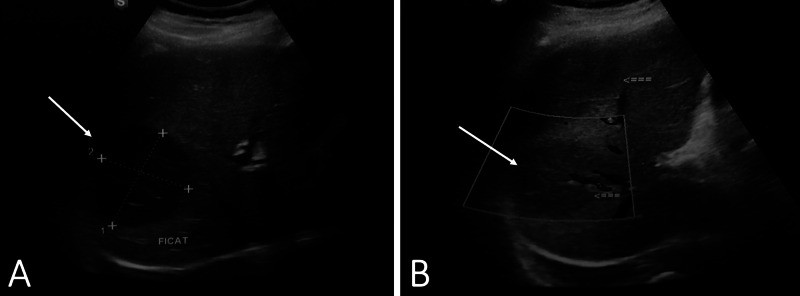
Ultrasonography of the liver showing masses (arrow) in the right (A) and left hepatic lobes (B).

On abdominal ultrasound, both left and right kidneys were of normal size with no calcifications. Hepatic ultrasonography revealed two lesions (Figure 2). The first mass was localized to the sixth and seventh segment of the right hepatic lobe, measuring 7.2 x 5.8 cm, and the second mass was localized to the sixth segment of the left hepatic lobe, measuring 9.8 x 6.8 cm. Both lesions were heterogenous, hypoechogenic, and presented with absent Doppler signs. Esophagogastroduodenoscopy and colonoscopy did not reveal any abnormalities, except mild reflux esophagitis. The workup for the chronic axonal neuropathy included ruling out vitamin deficiencies, infection (HIV, Borrelia), connective tissue diseases (Sjogren, systemic lupus erythematosus, rheumatoid arthritis), metabolic (diabetes), paraneoplastic (lung cancer), and endocrine diseases (hypothyroidism).

At this point, there was a high suspicion for chronic viral hepatitis-induced hepatocarcinoma leading to type III CG; however, the negative viral titers failed to corroborate this trail of thought. The absence of significant hematuria and proteinuria also excluded vasculitis as the cause of the AKI. Given the elevated urinary light chains a hematologic neoplasia with monoclonal gammopathy was, therefore, suspected as being the cause of the neuropathy and the cutaneous vasculitis.

**Figure 3:**
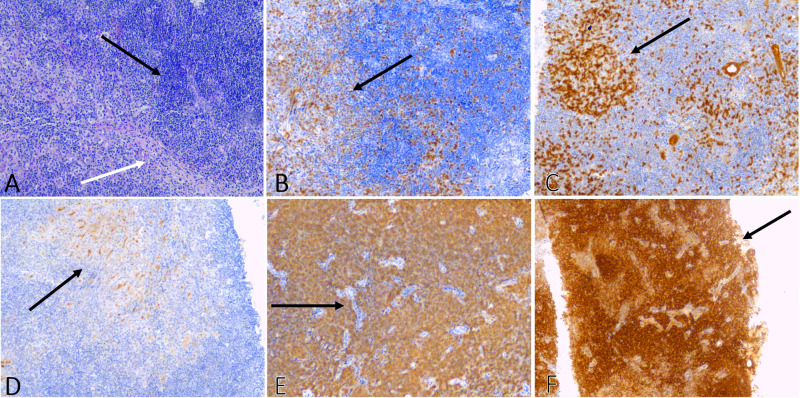
Histopathologic analysis of the liver biopsy: image A (H&E, x200) shows dense lymphocytic infiltrate (black arrow) with a vascularized fibrotic septum (white arrow); image B (IHC, x200) shows modest staining for CD3 (black arrow), corresponding to moderate T-cell infiltration; image C (IHC, x40) shows positive staining for CD5 (black arrow), a mantle cell lymphoma marker; image D (IHC, x200) shows modest staining for CD10 (black arrow), thus steering the diagnosis away from HCC, DLBCL, Burkitt lymphoma, and follicular lymphoma; image E (IHC, x200) shows intense staining for CD20, which highlights B-cell infiltration particularly around ducts hepatic ducts (black arrow); lastly, image F (IHC, x40) shows diffuse staining for BCL2 (black arrow). H&E: hematoxylin and eosin; CD: cluster of differentiation; IHC: immunohistochemistry; HCC: hepatocellular carcinoma; DLBCL: diffuse large B-cell lymphoma; BCL2: B-cell lymphoma 2.

The patient was started on diuretics (intermittent furosemide, 240 mg/day intravenous) and received plasmapharesis for three days. Pulsed therapy with prednisone (240 mg/day, five days total) was started on the third day of hospitalization and was replaced with methylprednisone on day nine. On day three, hepatic and dermal biopsies were also done - unfortunately, a renal biopsy could not be done at this time. On hepatic biopsy, the histopathologic and immunohistochemical findings demonstrated lymphoid cells that stained positive for cluster of differentiation (CD)3, CD5, CD10, CD20, B-cell lymphoma-2, among other markers (Figure 3). The biopsy results cemented the diagnosis of a primary hepatic MALT lymphoma in the absence of lymphoproliferative disease outside the liver. Serological investigation into the possibility of autoimmune hepatitis was warranted but not conducted due to the patient’s financial constraints.

**Figure 4:**
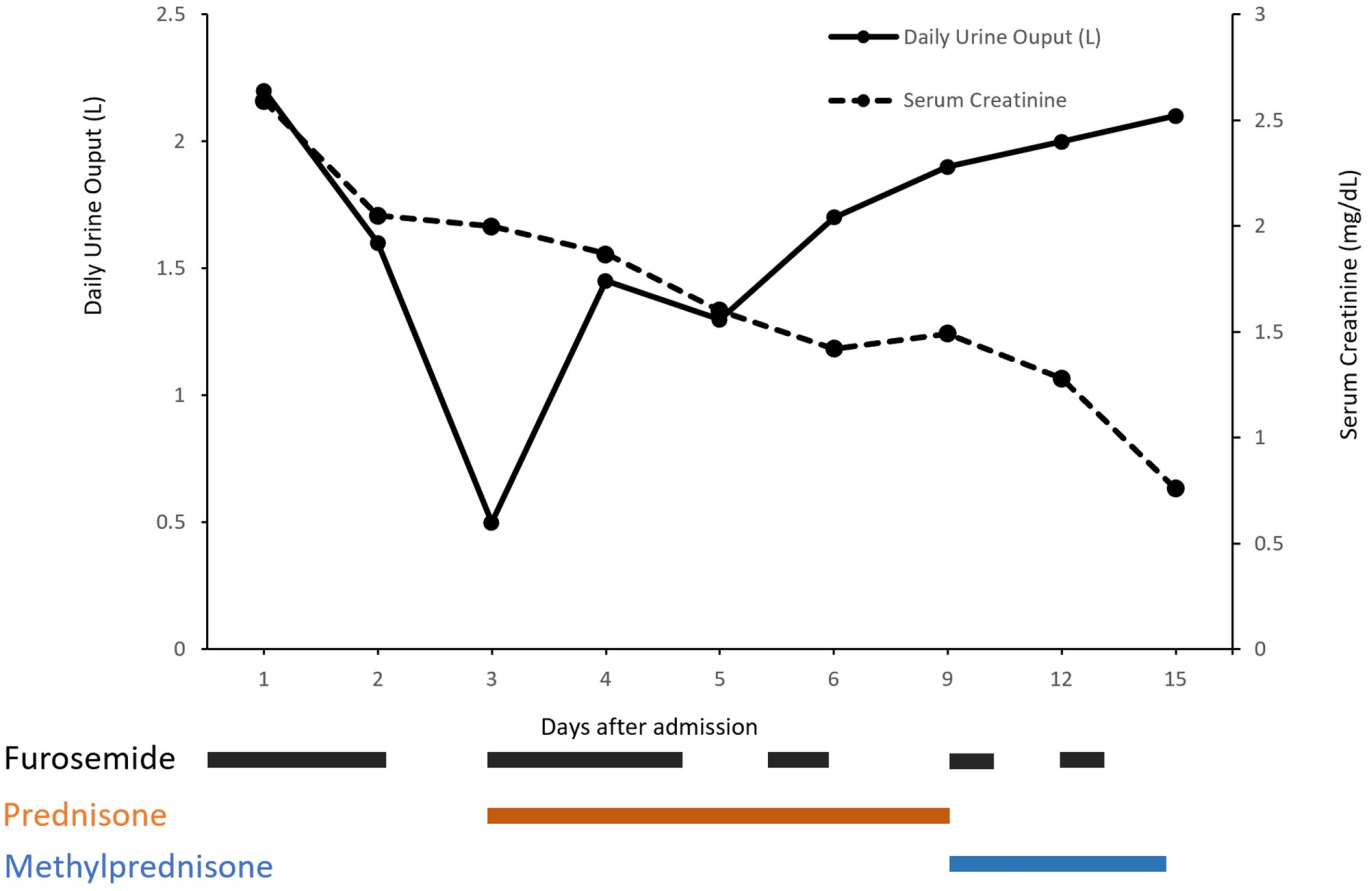
Evolution of the patient's renal function* and the accompanying therapy over 15 days of hospitalization at our department. *Glomerular filtration rate values were not included as renal function was not stable.

Throughout the hospitalization, renal function and cutaneous lesions improved under therapy with loop diuretics, high-dose glucocorticoids, and the evolution of renal function is summarized in Figure 4. Liver enzymes also improved and normalized to near normal values by the time of discharge. 

The patient was also referred to oncology and promptly started on rituximab, cyclophosphamide, doxorubicin, vincristine, and prednisolone (R-CHOP regimen). Due to the negative serology, the suspicion for an underlying chronic viral hepatitis infection was raised in hindsight, and polymerase chain reaction tests to assess for viral load were not conducted before the initiation of chemotherapy. Three months later the patient was re-admitted with fulminant hepatitis, which proved fatal. There was high viral load of HBV and hepatitis D virus, despite negative serology for the surface antigen. It was unclear at which point he contracted the infection at this time. 

## Discussion

We presented a patient with AKI, sensorimotor neuropathy, cutaneous ulcero-necrotic lesions secondary to type I CG that was caused by a primary hepatic MALT lymphoma. Néel et al. described the clinical characteristics of 36 patients with type I CG and the most common features of the disease [5]. Curiously, they identified 227 patients in whom type I cryoglobulins were detectable, but the majority of their patients (191) were asymptomatic, meaning approximately 85% of patients did not have any manifestations of this disease. The clinical features of type I CG from their study, and whether or not these findings were present in our patient, are summarized in Table 2. Cutaneous and vasomotor findings (defined as Raynaud phenomenon or paroxysmal acrocyanosis) were consistently the most common findings in symptomatic patients in their study.

**Table 2:** Common clinical features of symptomatic patients with type I CG. CG: cryoglobulinemia; Ig: immunoglobulin. *Severe skin lesions were defined by infarction, ulceration, and necrosis.

Signs and symptoms	Prevalence in IgG type I CG	Prevalence in IgM type I CG	Present in our patient
Sensorimotor neuropathy	36%	52%	Yes
Nephropathy	55%	20%	Yes
Vasomotor signs	18%	36%	No
Mild skin lesions (purpura)	64%	36%	No
Severe skin lesions*	55%	8%	Yes
Arthralgia	36%	12%	No

We maintain high index of suspicion that the cutaneous lesions of our patient are due to cryoglobulinemic vasculitis. With the associated symptoms of AKI, neuropathy, and hepatic lesions, four types of vasculitis per the Chapel Hill classification could be considered: cryoglobulinemic vasculitis, lupus vasculitis, hepatitis virus-associated, and cancer-associated vasculitis [6]. Negative viral serology and normal titers of anti-nuclear and anti-double stranded deoxyribonucleic acid (DNA) antibodies effectively rule out lupus and hepatitis virus-associated vasculitis. Although it has been described that many solid cancers (including lymphoma) can act as vasculitis-triggering factors, due to the clinical picture, elevated cryoglobulins, and statistical likelihood, cryoglobulinemic vasculitis is a more probable diagnosis [7].

To further explore the association between type I CG and renal involvement, a search of the PubMed and Scopus databases was conducted by using different combinations of keywords “type I cryoglobulinemia,” “cryoglobulinemic glomerulonephritis,” “clinical presentation,” and “clinical characteristics” to identify relevant studies. The keywords were identified via the medical subject heading (MeSH) terms or within the title or abstract. Case reports/series and studies that evaluated all types (type I with type II and III) of CG were excluded, as were studies that evaluated type I CG as caused by an isolated pathology (e.g., multiple myeloma). We identified five studies that fit per our selection strategy, and the results are presented in Table 3 [5,8-11].

**Table 3:** Summary of studies that described renal involvement in patients with type I CG. MPGN: membranoproliferative glomerulonephritis; GN: glomerulonephritis; NA: not applicable. *For some studies, results of all of the biopsies were not included.

Author and year	Total patients and mean age	Number of cases with renal involvement	Serum creatinine and proteinuria	Number of cases with renal involvement and conducted biopsy	Type of renal involvement*
Terrier et al., 2013 [9]	64 patients, 65.4 years	19	NA and 1.12 g/day	18	MPGN (17), GN with isolated C3 deposits (1)
Néel et al., 2014 [5]	36 patients, 63.0 years	11	3.55 mg/dL and 4 g/day	10	MPGN (7), thrombotic microangiopathy (2), lymphomatous infiltration (1)
Harel et al., 2014 [11]	64 patients, 62.0 years	13	NA and >1 g/day	9	MPGN (7), glomerular capillary congestion (1)
Sidana et al., 2017 [10]	102 patients, 59.0 years	14	NA	13	MPGN (9), proliferative GN (3), thrombotic microangiopathy (1)
Zhang et al., 2020 [8]	45 patients, 61.0 years	7	NA and >0.5 g/day	4	MPGN (3), endocapillary proliferative GN (1)

The studies were comprised of 311 patients with type I CG, 64 (20.6%) of which were reported to have renal involvement, consistent with other reports in literature [12]. A total of 54 (17.3%) patients who had renal involvement also underwent renal biopsy for diagnosis and evaluation of disease activity. Three studies (31 patients total) made the distinction between IgG and IgM isotypes of type I CG [5,8,11]. Some variables, like serum creatinine and proteinuria, were not uniformly defined by all studies. MPGN was the predominant type of renal involvement, followed by proliferative GN (and its subtypes, namely, endocapillary proliferative GN or C3 glomerulopathy) and is consistent with the literature, where MPGN is reported to account for 70%-90% of GN cases in patients with CG [13]. Furthermore, of these 311 patients, only three presented with AKI and nephrotic range proteinuria. The majority of patients presented with delayed disease and paucity of extrarenal manifestations, displaying the importance of regular monitoring of renal function to detect indolent renal function decline. Unfortunately, because a renal biopsy was not done, we can only speculate regarding the cause of the AKI in our patient - we believe the most likely cause is tubular obstruction by light chains, as cast nephropathy associated with a gastric IgM-secreting MALT lymphoma has been described [14]. Cryoglobulinemic MPGN is statistically more likely, but our patient lacked the hallmark features of overt hematuria and/or proteinuria, making it more difficult to argue for this diagnosis.

**Figure 5:**
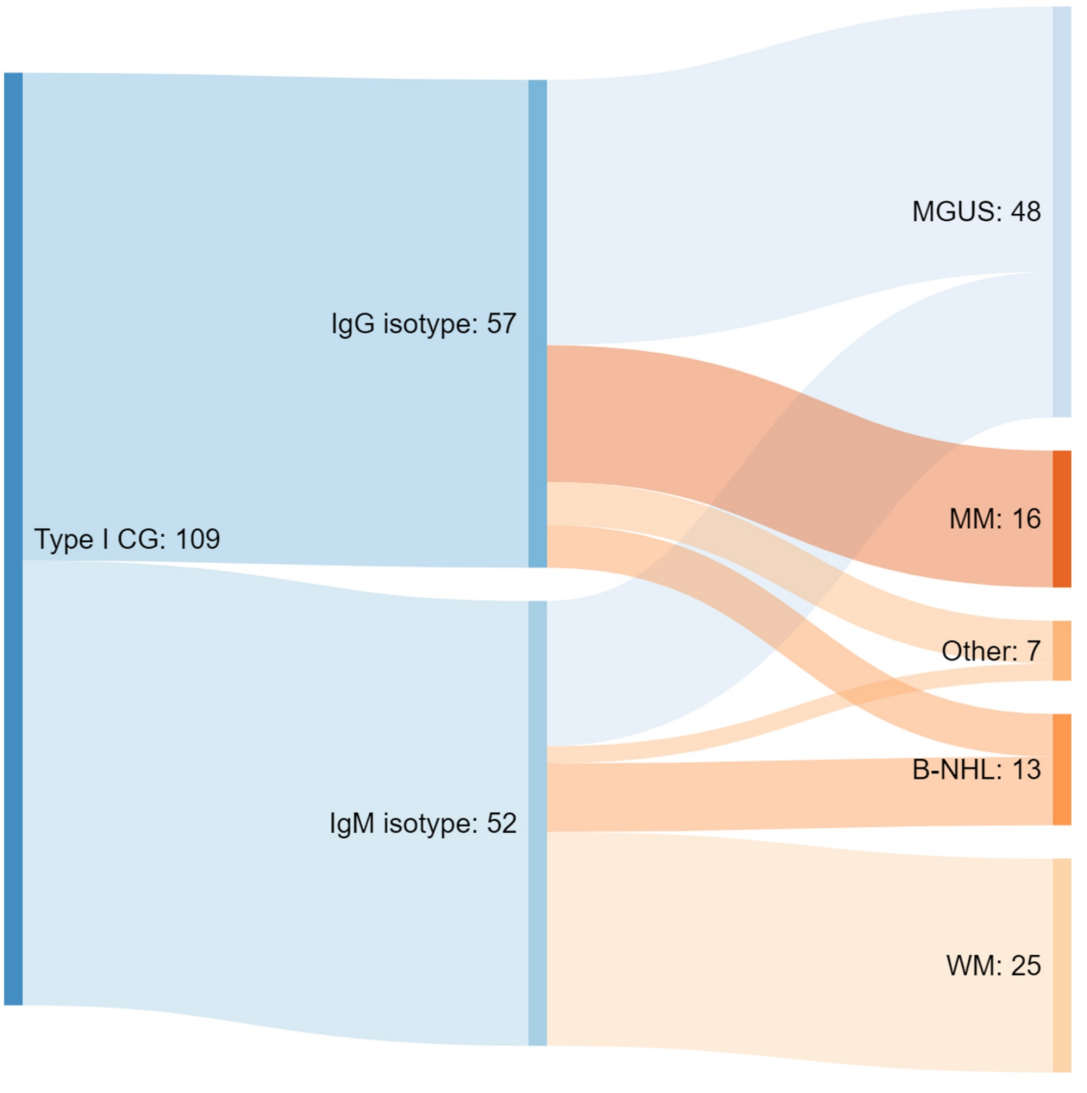
Etiologies of type I CG divided by predominent isotype. CG: cryoglobulinemia; IgG: immunoglobulin G; IgM: immunoglobulin M; MGUS: monoclonal gammopathy of undetermined significance; MM: multiple myeloma, B-NHL: B-cell non-Hogdkin lymphoma; WM: Waldenstrom macroglobulinemia. *Other causes included chronic lymphocytic leukemia, cold agglutinin disease, and amyloidosis.

The three most common lymphoproliferative causes for IgG type I CG are MGUS (n = 31), MM (n = 16), B-NHL (n = 5), while for IgM type I CG they are WM (n = 25), MGUS (n = 17), B-NHL (n = 8), and this information is summarized in Figure 5. These values were calculated from two studies, which divided their patients (109 total) according to the predominant Ig isotypes and reported the associated lymphoproliferative disorders discovered at diagnosis [8,11].

B-NHL is an uncommon cause of type I CG and, in our case, represents another unique aspect, being a MALT lymphoma localized to the liver that is extremely rare - only 67 cases have been reported in literature [15]. Furthermore, the diagnosis of MALT lymphoma was accidental, without directly or indirectly influencing the symptoms for which the patient presented to the hospital. Response to glucocorticoids in our patient was excellent, with serum creatinine improving from a peak of 2.59 mg/dL to 0.76 mg/dL over a two-week period. Estimated glomerular filtration rate also improved from 25 mL/min to 61 mL/min. Several cases of biopsy-proven cryoglobulinemic MPGN associated with MALT lymphoma have been described and authors noted similar recovery rates of renal function, although over a longer four-week hospital course [16,17]. The faster rate of recovery of renal function is the second reason why tubular obstruction can be considered as the cause of AKI.

Lastly, we would like to draw attention to the occult HBV infection that led to the death of the patient. Screening for the HBV surface antigen (HBsAg) was done at admission and subsequently on a monthly basis - it was repeatedly negative. The rates of occult HBV infection (i.e., HBsAg negative) were found to range from 6.1% to 11.0% in patients with B-NHL, thus posing a significant risk of reactivation during immunosuppressive treatment [18,19]. Elbedewy et al. noted a 6.94% reactivation rate of occult HBV in their patients upon initiation of chemotherapy for B-NHL, which negatively impacted survival [19]. The chronic inflammatory infiltrate on liver biopsy in our patient was a clue that the patient may have been positive for HBV. He was also not vaccinated for HBV but did not receive blood transfusions or underwent surgery. Although prophylactic treatment is not recommended, screening with quantitative HBV DNA in patients with lymphoma prior to chemotherapy may be warranted, especially if the immunosuppressive regimen involves biologic therapy [20].

## Conclusions

Renal involvement in patients with type I CG is relatively common, especially in the IgG isotype of the disease. The most common cause of renal impairment is MPGN with a gradual, indolent onset and a paucity of extrarenal manifestations - regular screening for renal function is important in this patient subset. In rare cases, such as ours, cryoglobulinemic MPGN can manifest as AKI, and other clinical findings, such as cutaneous lesions and peripheral sensorimotor neuropathy, should raise suspicion for the disease. Clinicians should expect acceptable recovery of renal function under treatment with glucocorticoids. Unfortunately, virtually all cases of type I CG are caused by an underlying lymphoproliferative disorder, and a search for a cause should promptly be started. If the diagnosis is a B-HNL, screening via quantitative HBV DNA may be warranted, even in context of negative HBsAg, to exclude the possibility of occult HBV reactivation and avoid fulminant hepatitis.
